# SARS-CoV-2 Specific Humoral Immune Responses after BNT162b2 Vaccination in Hospital Healthcare Workers

**DOI:** 10.3390/vaccines10122038

**Published:** 2022-11-29

**Authors:** Maryam Golshani, Ludmila Maffei Svobodová, Lubomír Štěpánek, Jan Zeman, Petra Nytrová, Helena Posová, Petra Petrásková, Olga Novotná, Michaela Nováková, Viktor Černý, Jiří Beneš, Libuše Kolářová, Martin Vokurka, Jiří Hrdý

**Affiliations:** 1 Institute of Immunology and Microbiology, First Faculty of Medicine, Charles University, 121 08 Prague, Czech Republic; 2 Institute of Biophysics and Informatics, First Faculty of Medicine, Charles University, 121 08 Prague, Czech Republic; 3 General University Hospital in Prague, 128 08 Prague, Czech Republic; 4 Institute of Pathological Physiology, First Faculty of Medicine, Charles University, 121 08 Prague, Czech Republic

**Keywords:** SARS-CoV-2, COVID-19, spike protein, mRNA vaccine, BNT162b2, antibody titer, ELISA

## Abstract

Background: COVID-19 pandemic has led to a loss of human life in millions and devastating socio-economic consequences worldwide. So far, vaccination is the most effective long-term strategy to control and prevent severe COVID-19 disease. The aim of the current study was to evaluate the humoral immune responses raised against the BNT162b2 vaccine in hospital healthcare workers. Methods: Total number of 173 healthcare workers enrolled in the study. Their blood samples were collected in three different time intervals after the second SARS-CoV-2 vaccination and evaluated by the ELISA method to detect anti-spike protein IgM and IgG antibodies. The baseline characteristics of all participants were collected using questionnaires and were evaluated for finding any significant data. Results: Our results demonstrated that the levels of antibodies were higher in the young group (21–30 years old) and also among male participants. Moreover, the highest levels of antibodies were detected from the group that received the third shot vaccination. Conclusions: Our results indicate that age, gender and third-dose vaccination can affect the levels of humoral immune responses against the BNT162b2 vaccine in healthcare workers.

## 1. Introduction

Severe acute respiratory syndrome coronavirus (SARS-CoV)-2 was identified in mid-December 2019 in the city of Wuhan of Hubei Province, China, and rapidly spread across the world [1,2,3,4,5,6]. SARS-CoV-2 is an enveloped, single-stranded positive-sense RNA beta coronavirus from the same family as SARS-CoV and Middle East respiratory syndrome coronavirus (MERS-CoV). On 11 March 2020, the World Health Organization (WHO) officially declared the COVID-19 disease as a pandemic [1,2,5,6,7,8,9,10]. From the beginning of the pandemic until the time of writing this manuscript (20 October 2022), in total, 631,663,217 confirmed cases and 6,577,758 death cases have been reported in 224 countries [11]. So far, vaccination is the most effective long-term strategy to control and prevent severe COVID-19 disease. Presently, more than 100 vaccines have been tested/are being tested and some of them have received conditional authorization from the Food and Drug Administration (FDA) and European Union (EU) for commercial use [3,4,5,8,12,13]. 

The COVID-19 vaccines authorized in the European Union (EU) following evaluation by the European Medicines Agency (EMA) are as follows: BNT162b2 (developed by BioNTech and Pfizer), mRNA-1273 (developed by Moderna and NIAID), AZD1222 (ChAdOx1) (developed by Oxford university and AstraZeneca AB), JNJ-78436735 (Ad26.COV2.S) (developed by Janssen Pharmaceuticals) and NVX-CoV2373 (developed by Novavax) [14,15].

On 1 March 2020, the first three cases of COVID-19 disease were identified in the Czech Republic (CR) with 10,739,703 total population [16]. Until today, a total of 4,147,736 positive cases and 41,454 deaths have been officially reported in CR [17,18]. On 15 January 2021 and 26 January 2021, CR launched vaccination against COVID-19 disease for persons 80+ and healthcare professionals, respectively. On 29 November 2021, a booster dose vaccination was launched for people over the age of 60 and people at risk after just 5 months from the second dose [19,20]. At the end of January 2022, 61% of the population was fully vaccinated and 65% had at least received the first dose of vaccination [19,20]. There are the following four approved COVID-19 vaccines available in the Czech Republic: BNT162b2, mRNA-1273, JNJ-78436735, AZD1222 [14]. Infection of SARS-CoV-2 activates innate immune responses as well as antigen-specific cytotoxic T-cells directed against the virus and also B-cell responses with the ultimate production of neutralizing antibodies [21]. These antibodies predominantly target the N protein and the receptor-binding domain (RBD) of the spike protein. Therefore, most COVID-19 vaccines typically target the spike protein of the SARS-CoV-2 virus [4].

BNT162b2 (also known as Comirnaty) is a lipid nanoparticle (LNP)—encapsulated mRNA vaccine encoding the modified SARS-CoV-2 full-length spike (two proline mutations are applied to lock it in the prefusion conformation) [9]. In Phase III trials, administration of 2 dosages of BNT162b2 (30 μg) vaccine in persons 16 years of age or older showed 95% efficacy. The overall frequency of the side effects of myocarditis and pericarditis has been determined by EMAs Pharmacovigilance Risk Assessment Committee (PRAC) to be very rare (one case in 1 million population). The risk has been confirmed to be highest in young males [3,4,9,15]. 

mRNA vaccines are at the forefront of pandemic-response vaccine development due to their rapid, flexible and efficient strategy in immunogen design and manufacturing [9]. The current COVID-19 mRNA vaccines contain purified, in vitro-transcribed single-stranded mRNA encapsulated in LNPs. Modification of mRNA nucleotides reduces its binding to Toll-like receptor (TLR) and immune sensors, resulting in limited production of type I interferon that has an inhibitory effect on the cellular translation of the immunogen. The LNP carrier protects the mRNA, facilitates its target delivery to the lymphatics and promotes protein translation in the lymph nodes. Eventually, in the lymph nodes, LNP is engulfed by dendritic cells (DC), where the immunogen will be produced and presented to T cells for activation of the adaptive immune response [22]. Studies demonstrate that a complete intramuscular vaccination with BNT162b2 induces strong systemic humoral responses (anti-S1 and neutralizing antibodies) detectable in serum samples but a weak mucosal immune response characterized by low IgG and IgA antibodies to the spike protein and the RBD in saliva [23]. The results of a study performed by Turner et al. show that mRNA vaccines induce persistent germinal center B cell responses, which enable the generation of robust humoral immunity. The majority of spike protein monoclonal antibody clones derived from germinal center B cells lymph nodes 12 weeks after the booster vaccination predominantly target the RBD, while others bind to the N-terminal domain or to epitopes shared with the spike proteins of the human beta coronaviruses OC43 and HKU1 [24].

The aim of the present study was to evaluate the humoral immune responses upon vaccination with BNT162b2 in healthcare workers in Prague, Czech Republic.

## 2. Materials and Methods

### 2.1. Subjects

Total number of 173 healthcare workers from the General University Hospital in Prague have been enrolled in the study after signing the written informed consent. Anamnestic data including age, gender, BMI, occupation, time of vaccination, status of third dose vaccination, positivity for SARS-CoV-2 or the course of history of COVID-19 infection and contact with the SARS-CoV-2 positive cases were collected from questionnaire filled during every blood collection. The study was approved by Ethics Committee of the General University Hospital in Prague, Prague, Czech Republic (43/21 S-IV). Peripheral blood samples were collected in the following three different time intervals: 2–3, 4–6 and 6–8 months after the full vaccination (two doses of mRNA vaccine BNT162b2), at the General University Hospital in Prague. Detailed baseline characteristics of the study group are presented in Table 1, Table 2 and Table 3.

### 2.2. Antibody Detection

Sera were collected from all blood samples and were stored at −20 °C for further experiments. Enzyme-Linked Immunosorbent Assay (ELISA) was used to evaluate the capacity of BNT162b2 vaccine to elicit humoral responses. The concentration of anti-spike protein-specific antibodies was measured using commercially available ELISA kits for IgG (cat. no. COR-QNS-IGG-S1, Matriks Biotek, Ankara, Turkey) and IgM (cat. no. COR-QNS-IGM-S1, Matriks Biotek, Ankara, Turkey) antibodies according to the manufacturer instruction. The standard calibration curve was created, and data were analyzed using software KIM (Schoeller Instruments, Prague, Czech Republic).

### 2.3. Statistical Analysis

GraphPad Prism version 8 software (GraphPad, San Diego, CA, USA) and Microsoft Excel software (Pivot tables) were used for graphical and statistical evaluation and processing of the data obtained. Continuous variables were expressed as mean and standard error of the mean (SEM). Besides descriptive statistics performed using contingency and frequency tables, Statistical significance was determined using non-parametric one-way analysis of variance followed by Dunn multiple comparison post hoc tests and two-way ANOVA with Bonferroni post hoc tests. Statistical outputs with *p* values ≤ 0.05 were considered statistically significant.

## 3. Results

### 3.1. Participant Characteristics

Among a total number of 173 healthcare worker volunteers enrolled in our study, most were in the age group 41–50 years old (24%). In total, 61.8 percent were females; among them, 13.3 percent were in the age group 41–50 years old. In total, 71.9% of the attendants had a high BMI between 18.4 and 28.4; most of them belonged to the age group 21–30 years old (15%). All participants received two dosages of the BNT162b2 vaccine; among them, only 60.1% of the participants (104/173) received the booster shot (third dose). The time distribution of vaccination against SARS-CoV-2 is shown in Supplementary Table S1. The vaccination was launched in December 2020, and most participants received their first (42.8%) or second shot (32.9%) in January 2021. Most of the booster-vaccinated participants received their third shot in November 2021 (31.7%) and the third blood collection was performed 2–11 weeks after receiving their third vaccination dose. The percentage of booster-vaccinated cases was higher in female participants (68.3%). Most participants (122/167) reported no history of COVID-19 and only 48 cases out of 167 attendants (28.7%) reported a history of laboratory-confirmed COVID-19 infection; from them, 95.6% were infected before the first shot, 4.2% were infected between first and second shot and 6.1% were infected between the second and third vaccine dose. Most of the cases with a history of SARS-CoV-2 infection were symptomatic (Table 1). Fatigue (36/42) followed by anosmia (28/42) and headache (28/42) were the most common symptoms in infected participants (Table 2). 

According to Table 4, most of the participants were from the nurse occupation group (18.2%), who were also the most booster-vaccinated cases (23.3%) in comparison to other occupation groups. However, nurses had the highest number of cases with a history of symptomatic COVID-19 infection as well. 

Regarding the comorbidities, obesity was the most common comorbidity (19.8%), followed by allergy (16.2%). Cases with a high BMI (between 30 and 39 kg/m^2^) had the highest rate of COVID-19 infection (20%) (Table 4).

Finally, 52.1% of the healthcare workers have taken regular medication; most of them were in the age group 61–70 (24.1%) (Table 2).

### 3.2. Humoral Immune Responses

**Figure 1:**
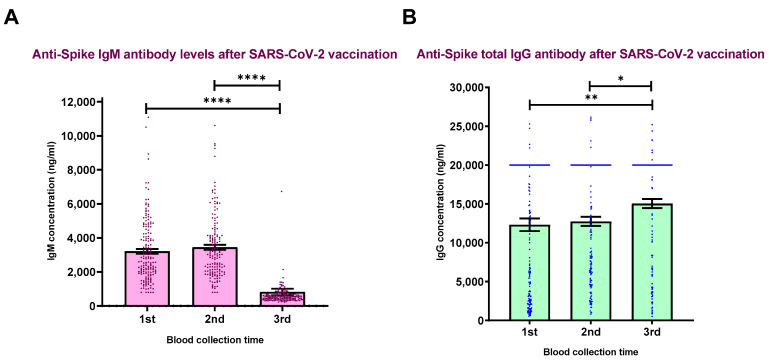
Levels of anti-spike IgM (**A**) and IgG antibodies (**B**) measured after three blood collection intervals. Concentration of specific antibodies against spike protein in peripheral blood of healthcare workers has been detected by commercially available ELISA kits (anti-spike IgM—(**A**); anti-spike IgG—(**B**)). * *p* value 0.04, ** *p* value 0.009 and **** *p* < 0.0001.

Blood samples were collected at the following three different intervals: 2–3 months, 4–6 months and 6–8 months upon the full vaccination. The third blood collection was performed 2–11 weeks after the booster dose. Analyzing the antibody levels in sera samples showed that the levels of anti-spike IgM antibody decreased gradually and reached the lowest level after the third vaccination (Figure 1A). In contrast, the level of anti-spike IgG antibody remained stable 5 months after the vaccination (*p* > 0.05) and reached its highest level after the third vaccination as the was a statistically significant difference between the levels of IgG from last blood collection and two other blood collection times (*p* < 0.05) (Figure 1B). Therefore, the highest antibody levels (16,000–26,000 ng/mL) are only detected in booster-vaccinated volunteers (Table 5).

The highest number of participants raised anti-spike IgG levels between 16,000 and 21,000 ng/mL (40.5%); from these, 27.1% were in the age group 21–30, 46.9% were men and 34.4% were women; 26% had a history of COVID infection with or without symptoms; 24.7% had symptoms after vaccination rather than injection site pain (Table 5). 

The highest number of participants raised anti-spike IgM levels between 2000 and 3000 ng/mL (40.5%) from these 21.4% were in the age range 30–50 years old, 38.3% were men and 42.4% were women, 26% the history of COVID infection with or without symptoms, 21.6% had symptoms after vaccination rather than injection site pain (Table 5).

Participants without COVID-19 infection raise anti-spike IgG levels between 11,000 and 21,000 ng/mL (41.7%) and anti-spike IgM levels between 2000 and 3000 ng/mL (35.7%). In total, 40.5% of fully vaccinated cases and 40.4% of cases that received a third shot raised anti-spike IgG levels between 16,000 and 21,000 ng/mL (Table 5).

**Figure 2:**
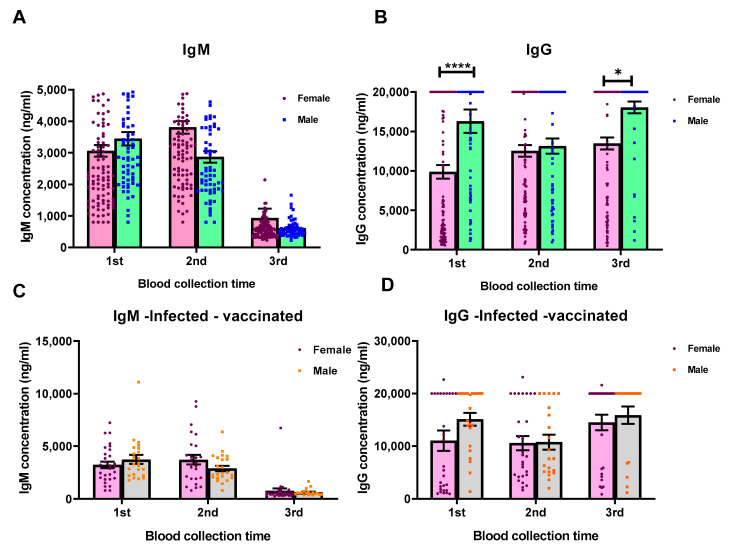
Comparison of the anti-spike antibody levels between male and female participants. (**A**) Anti-spike IgM levels in all male (66/173) and female (107/173) participants, (**B**) anti-spike IgG levels in all male and female participants, (**C**) anti-spike IgM levels in male (23/173) and female (29/173) participants without the history of laboratory-confirmed infection or third vaccination, and (**D**) anti-spike IgG levels in male and female participants without the history of laboratory-confirmed infection or third vaccination. * *p* value 0.02 and **** *p* value < 0.0001.

The difference in levels of anti-spike IgG antibodies raised by males in comparison to those achieved by females was statistically significant (*p* < 0.05). All male participants (including the third dose vaccinated or infected cases) showed higher IgG antibody levels than all female participants did (Figure 2B).

## 4. Discussion

The aim of the current study was to evaluate the humoral immune responses raised against the SARS-CoV-2 BNT162b2 mRNA vaccine in healthcare workers from the General Hospital in Prague. According to our results, not all the fully vaccinated healthcare workers attended for the third shot, as only 60.1 percent of the participants received the third dose of vaccination against the SARS-CoV-2 virus. This can be due to the following reasons: (i) the decrease in the number of severe COVID-19 cases that affected people’s interest in attending for the third shot, (ii) getting infected between the second and third shot, or (iii) experience of severe side effects after the second injection. Most participants reported no history of COVID-19 infection, although the Czech Republic was one of the European countries with the highest number of COVID-19 cases (17). This contrast can be explained by the fact that many positive cases may not be detected using routing rapid screening tests or they can be simply asymptomatic. Moreover, vaccination can protect people against infection/severe infection as among cases with a history of infection, 96 percent of participants reported being infected before the first vaccination shot. More than half of the infected participants are females, and most of these females experienced COVID-19 symptoms during their infection.

Regarding the distribution of the occupations (Table 3), the nurses represent the highest proportion of the participants. Although all nurses are booster-vaccinated, the highest number of symptomatic COVID-19-infected cases belong to this group. This can be explained by the fact that nurses are at a high risk of contact with COVID-19-infected patients. This data can also be confirmed by our results from Table 3, which show that among all occupational groups, nurses had the highest contact rate with COVID-19-infected patients.

Regarding the effect of comorbidities on the rate of infection, cases with a high BMI had the highest number of COVID-19 infections. This group of participants also shows the highest percentages of side effects after the second and third vaccinations. In contrary with our result, Iguacel et al. showed that obesity is not associated with severe adverse effects after COVID-19 vaccination as non-overweighted individuals have a higher risk of fever ≥38 °C, vomiting, diarrhea and chills compared to overweighted ones [25]. Moreover, Menni et al. could not find any significant link between obesity and COVID-19 vaccine side effects [26]. However, the results of the previous studies performed on the other vaccines indicate that cases with obesity show more severe side effects than the non-overweighted cases [27,28]. It is important to emphasize that additional confoundings such as different ethnicity, socio-economical status, quality of medical care, or type of questionnaire can contribute to a contradictory results published. Allergic participants show the highest percentages of side effects after the second and third vaccinations, as well. Generally, an incidence of anaphylaxis rate of 2.5–4.7 cases per million doses has been estimated for SARS-CoV-2 mRNA vaccines, which is higher than that associated with other vaccines [29]. Several previous studies also indicated the risk of allergic reactions upon SARS-CoV-2 vaccination. Shavit and his colleagues showed that the rate of allergic reactions to the BNT162b2 vaccine is higher among patients with allergies, particularly among cases with a history of high-risk allergies [30]. Regula et al. reported cases with immediate hypersensitivity reactions after vaccination with BNT162b2 or mRNA-1273 mRNA vaccines. The authors suggested that the graded dosing of these vaccines would be safe, efficacious and useful for treating these individuals with allergies [29]. In a systematic review and meta-analysis study performed by Chu and his colleagues, among 22 studies on SARS-CoV-2 mRNA vaccines, 1366 individuals showed immediate allergic reactions to their first vaccination. However, only 4 cases out of 1366 patients had a severe immediate reaction after the second shot [31]. According to Kaplan et al., the after-vaccination anaphylaxis rate was 40.6 cases per million and all cases were females [32]. It is currently hypothesized that polyethylene glycol (PEG) can be the cause of allergic reactions to SARS-CoV-2 mRNA vaccines and in a number of studies, patients were tested for the presence of pre-existing anti-PEG IgE antibodies [33,34,35,36]. However, Kaplan and his colleagues found that most severe postvaccination allergic symptoms are not caused by hypersensitivity to PEG [32]. In the letter to the editor published by Herman et al., the authors suggested that TX-100 may be an unrecognized contributor to allergic reactions to vaccines, especially in cases of uncertainty, and further exploration of this potential allergen may be required [37]. According to our results, none of the participants, including the ones with allergies, showed severe allergic reactions upon first, second or third vaccination with the mRNA BNT162b2 vaccine.

Evaluation of the anti-spike IgG antibodies demonstrates that the level of antibodies elevated to its peak level after the second shot, stayed almost at the same level for 4–6 months and boosted again after the third vaccination shot (Figure 1B). Accordingly, there is a statistically significant difference between the level of IgG antibody after the first blood collection and the other two intervals (*p* < 0.05). Our results show that the highest antibody levels are only detected in booster-vaccinated volunteers. These results are consistent with the findings of the study performed by Busa et al., which indicated the advantage of third-dose mRNA vaccination on boosting IgG and IgA antibody levels as well as the lifespan of memory B and T cells, suggesting that a booster dose can enhance protection against COVID-19 infection [38]. In the study performed by Franzese et al., mRNA BNT162b2 vaccinated healthcare workers were monitored for one month upon the third dose of vaccination. The authors found that the anti-spike antibody titers decreased after the second injection but rose after the third vaccination [39]. Bonnet and his colleagues also evaluated anti-RBD antibody titers at different intervals upon vaccination. Their results show that the levels of the antibodies decreased between 3 and 6 months after vaccination [40]. Takeuchi et al. concluded that the antibody titers show a time-dependent reduction 3 months after vaccination [41]. Morgiel et al. show that humoral responses were detectable 7–9 months after vaccination but almost fell down by 90% [42]. The authors indicate that past SARS-CoV-2 infection is a factor associated with higher immunogenicity. According to our results, this factor did not affect the immunogenicity against the mRNA vaccine, as around 40 percent of cases raising the highest IgG levels had a history of COVID-19 infection. Furthermore, the majority of the volunteers raised levels of IgG antibodies in the range of 16,000–21,000 ng/mL; among them, there is no statistically significant difference between the non-infected and infected individuals (*p* > 0.05).

Another finding of our study indicates that immune responses against SARS-CoV-2 are higher in younger cases since the levels of anti-spike IgG antibodies in about half of the 21–30 years-old age group were elevated to 16,000–21,000 ng/mL (*p* < 0.05). These results are consistent with the results of the previous studies showing that, although all age group participants produce specific IgG antibody titers against the SARS-CoV-2 spike protein, titers were significantly lower in elderly volunteers [41,43,44,45,46]. However, Jimenez and his colleagues also studied the current strategies being used for the development of COVID-19 vaccines, and they concluded that different vaccines may be protective for different age groups within the population, depending on the strategy used for their design [44].

Regarding the differences in physiology, genetic background, TLR pathway response and microbiome, generally, males and females develop different responses to vaccines. Differences in vaccine uptake and subsequent adverse effects have been observed in males and females. Therefore, it should be considered in vaccine formulation and dosage prescribed for men and women [47]. According to the previously published studies, the efficacy of mRNA COVID-19 vaccines is higher in men than women, as BNT162b2 and mRNA-1273 are 96.4% and 95.4% effective in men and 93.7% and 93.1% effective in women, respectively [9,12]. In the current study, regarding the corresponding percentage, higher specific antibodies were detected from male participants than female participants. The IgG concentrations rose significantly after the first and third vaccinations in men than in women (*p* < 0.05). This can be explained by the higher humoral responses against the COVID-19 vaccine in male participants. These results are consistent with the findings of Bonnet et al., who presented higher anti-RBD antibodies in males than females [40]. On contrary, there are studies indicating that generally females develop higher antibody responses and report more adverse reactions following vaccination including SARS-CoV-2 vaccines [41,46,48,49]. In order to explain the reason, Ciarambino et al. suggest the role of the sex hormone testosterone in the reduction of vaccination immune responses and depression of cytokine responses in males, resulting in a higher chance of widespread COVID-19 infection in men [50].

Another explanation for higher antibody levels in males (in the present study) may be the higher number of female participants than males in our study, which can generally affect the statistical analysis results (rate of females to males = 2). Moreover, as Figure 2 shows, after omitting the booster-vaccinated and infected volunteers from the groups, the levels of antibodies were not significantly different between the male and female groups (*p* > 0.05).

Limitations of our study include the prolonged time interval between the full vaccination (completed second dose) and the first blood collection, which could not cover the antibody peak levels occurring generally two weeks after vaccination in all participants. The reason for this issue was the distributed vaccination time among the participants, which made it quite challenging to keep the precise intervals for blood collection.

## 5. Conclusions

According to our results, age, sex and third shot vaccination are among the factors affecting the humoral immune responses against the SARS-CoV-2 BNT162b2 vaccine. Young participants (age group 21–30), males and volunteers who received their third vaccine shot showed higher anti-spike IgG antibody levels.

## Figures and Tables

**Table 1:** Distribution of baseline characteristics, vaccination status and COVID-19 infection history among healthcare workers [quantitative data are presented as number (%)].

Age Groups	Number (%)	3rd BNT162b2 Vaccine Dose		Gender (n = 173)		COVID-19 Infection (N = 48)		BMI kg/m^2^ (N = 167)				Time of COVID-19 Infection Regarding the Vaccination (N = 48)			Regular Medication (n = 167)	
						Symptoms										
		YES	No	Female	Male	Yes	No	18.4–28.4	28.4–38.4	38.4–48.4	48.4–58.4	Before 1st Dose	Between 1st and 2d Dose	Between 2d and 3d Dose	Yes	No
<21	7 (0.6)	6 (5.8)	1 (1.4)	5 (4.6)	2 (3)	-	-	1 (0.6)	-	-	-	1 (2.3)	-		1 (1.1)	-
21–30	32 (19.2)	13 (12.5)	17 (25)	21 (19.6)	11 (16.7)	8 (17.8)	2 (66.7)	25 (15)	5 (11.6)	2 (50)		10 (23.3)	-	1 (33.3)	10 (11.5)	22 (27.5)
31–40	30 (18)	15 (14.4)	14 (20.6)	18 (16.8)	12 (18.2)	10 (22.2)	1 (33.3)	23 (14)	5 (11.6)	1 (25)	1 (100)	9 (20.9)	2 (100)	1 (33.3)	12 (13.8)	18 (22.5)
41–50	40 (23.9)	27 (25.9)	13 (19.1)	23 (21.5)	17 (25.8)	11 (24.4)	-	29 (17.4)	11 (25.6)	-	-	11 (25.6)	-		20 (22.9)	20 (25)
51–60	30 (17.9)	22 (21.2)	9 (13.4)	19 (17.8)	11 (16.7)	7 (15.5)	-	19 (11.4)	10 (23.3)	1 (25)	-	7 (16.3)	-		14 (16.1)	16 (20)
61–70	24 (14.4)	16 (15.4)	9 (13.4)	15 (14)	9 (13.6)	5 (11.1)	-	15 (9)	9 (20.9)	-	-	5 (11.6)	-	1 (33.3)	21 (24.1)	3 (3.8)
>71	11 (5.9)	5 (4.8)	6 (8.8)	6 (5.6)	4 (6.1)	1 (2.2)	-	8 (4.8)	2 (4.7)	-	-	1 (2.3)	-		9 (10.3)	1 (1.25)
Total	173	104 (60.5)	68 (39.5)	107 (61.8)	66 (38.2)	45 (93.8)	3 (6.3)	120 (71.9)	43 (25.7)	4 (2.4)	1 (0.6)	43 (89.6)	2 (4.2)	3 (6.3)	87 (52.1)	80 (47.9)

**Table 2:** Presentation of the COVID-19 symptoms in healthcare workers with the history of laboratory-confirmed COVID-19 infection.

Symptom	No. *	Percentage **
Fatigue	36	87.8
Anosmia	28	68.3
Headache	28	68.3
Fever	23	56.1
Anorexia	22	53.7
Myalgia	18	43.9
Dry cough	17	41.5
Rhinorrhea	16	39
Arthralgia	15	36.6
Backache	13	31.7
Diarrhea	7	17.1
Nausea	7	17.1
Productive cough	4	9.8
Nausea	3	7.3
Vomiting	3	7.3
Ageusia	2	4.9
Other ***	8	19.2

* Total number of attendants with symptoms are 42, ** percentages do not sum to 100% because each person may have more than one symptom, *** sweating, chills, faint, chest pain and distress, epigastric pain, groin pain, swollen LNs, dizziness.

**Table 3:** Prevalence of SARS-CoV-2 infection and third dose vaccination status in different genders and occupations [Quantitative data are presented as number (%)].

Gender	Number (%)	3rd BNT162b2 Vaccine Dose (N = 172)		Contact with COVID-19 Positives		COVID-19 Infection (N = 170)		COVID-19 Symptoms (N = 48)	
		No	Yes	No	Yes	No	Yes	No	Yes
Female	107 (61.8)	35 (51.5)	71 (68.3)	78 (64.5)	25 (54.3)	78 (63.9)	27 (56.3)	2 (66.7)	25 (55.6)
Male	66 (38.2)	33 (48.5)	33 (31.7)	43 (35.5)	21 (49.7)	44 (37.1)	21 (43.8)	1 (33.3)	20 (44.4)
Total	173	68 (9.5)	104 (60.5)	121 (72.5)	46 (27.5)	122 (71.8)	48 (28.2)	3 (6.7)	45 (93.8)
Occupation									
Nurse	31 (18.2)	7 (10.4)	24 (23.3)	7 (10.4)	24 (23.3)	16 (13.2)	13 (28.3)	19 (16.1)	10 (20.8)
Doctor	24 (14.1)	6 (8.9)	18 (17.5)	6 (8.9)	18 (17.5)	13 (11.1)	11 (23.9)	19 (16.1)	5 (10.4)
Administrative/office staff	25 (14.7)	13 (19.4)	11 (10.7)	13 (19.4)	10	22 (18.6)	2 (4.2)	18 (15.3)	6 (12.5)
Laboratory staff	12 (7.1)	6 (8.9)	6 (5.8)	6 (8.9)	6 (5.8)	9 (7.7)	3 (6.5)	9 (7.6)	3 (6.3)
Medical assistant	10 (5.9)	5 (7.5)	5 (4.9)	5 (7.5)	5 (4.9)	8 (6.8)	2 (4.3)	10 (8.4)	-
Medical specialist	10 (5.9)	4 (5.9)	6 (5.8)	4 (5.9)	6 (5.8)	7 (5.9)	3 (6.5)	5 (4.2)	5 (10.4)
Head of Departemnt	8 (4.7)	4 (5.9)	4 (3.9)	4 (5.9)	4 (3.9)	7 (5.9)	1 (2.2)	7 (5.9)	1 (2.1)
Sanitar	6 (3.5)	2 (2.9)	4 (3.9)	2 (2.9)	4 (3.9)	2 (1.7)	4 (8.7)	4 (3.4)	2 (4.2)
Professor/Lecturer	5 (2.9)	3 (4.5)	2 (1.9)	3 (4.5)	2 (1.9)	4 (3.4)	1 (2.1)	4 (3.4)	1 (2.1)
IT	4 (2.4)	1 (1.5)	3 (2.9)	1 (1.5)	3 (2.9)	4 (3.4)	-	2 (1.7)	2 (4.2)
Midwife	3 (1.8)	1 (1.5)	2 (1.9)	1 (1.5)	2 (1.9)	3 (2.6)		2 (1.7)	1 (2.1)
Pharmacist	3 (1.8)	1 (1.5)	2 (1.9)	1 (1.5)	2 (1.9)	2 (1.7)	1 (2.1)	2 (1.7)	1 (2.1)
Dentist	2 (1.2)	-	2 (1.9)	-	2 (1.9)	2 (1.7)		1 (9.1)	1 (2.1)
Physiotherapist	2 (1.2)	-	2 (1.9)	-	2 (1.9)	-	2 (4.2)	-	2 (4.2)
Researcher	2 (1.2)	-	2 (1.9)	-	2 (1.9)	2 (1.7)		2 (1.7)	-
Student	2 (1.2)	2 (2.9)	-	2 (2.9)	-	2 (1.7)		2 (1.7)	-
Psychologist	1 (0.6)	1 (1.5)	-	1 (1.5)	-	1 (0.9)		1 (9.1)	-
Nutritionist	1 (0.6)	-	1 (0.9)	-	1 (0.9)		1	-	1 (2.1)
Other	20 (11.8)	11 (16.4)	9 (8.7)	11 (16.4)	8 (7.8)	14 (11.9)	4	11 (9.3)	7 (14.6)
Total	170	67	103	67	102	118	48	118	48

**Table 4:** Evaluation of the vaccination status and the history of SARS-CoV-2 infection in participants with comorbidities [quantitative data are presented as number (%)].

Comorbidities	Number (%)	Contact with COVID-19 Positives	COVID-19 Infection-Symptoms			Post-Full Vaccination Symptoms *	3rd BNT162b2 Vaccine Dose		
			No-No	Yes-Yes	Yes-No		Yes	No	Post-Vaccination Symptoms
Obesity (BMI 30–39 kg/m^2^)	22 (19.8)	5 (25)	15 (20.3)	7 (20)	-	4 (22.2)	12 (16.4)	10 (26.3)	5 (31.3)
Allergy	18 (16.2)	3 (15)	12 (16.2)	6 (17.4)	-	7 (38.9)	13 (17.8)	5 (13.2)	4 (25)
Gastrointestinal diseases	12 (10.8)	4 (20)	7 (9.5)	5 (14.3)	1 (50)	2 (11.1)	8 (10.9)	4 (10.5)	1 (6.3)
Blood factors diseases	10 (9)	3 (15)	9 (12.2)	1 (2.9)	-	-	4 (5.5)	6 (15.8)	1 (6.3)
Hypercholerterolemia	8 (7.2)	1 (5)	7 (9.5)	-	-	1 (5.6)	7 (9.6)	1 (2.6)	1 (6.3)
Neurological/Psychological diseases	7 (6.3)	2 (10)	5 (6.8)	2 (5.7)	-	-	7 (9.6)	-	1 (6.3)
Chronic heart disease	6 (5.4)	-	5 (6.8)	-	1 (50)	-	3 (4.1)	3 (7.9)	-
Palmunary diseases	3 (2.7)	-	3 (4.1)	-	-	-	1 (1.4)	2 (5.3)	-
Surgery	3 (2.7)	-	3 (4.1)	-	-	-	1 (1.4)	2 (5.3)	-
Immunosupression/immunodeficiency	2 (1.8)	-	2 (2.7)	-	-	-	2 (2.8)	-	-
Asthma	2 (1.8)	-	2 (2.7)	-	-	-	1 (1.4)	1 (2.6)	-
Osteo disorders	2 (1.8)	-	1 (1.4)	1 (2.9)	-	1 (5.6)	2 (2.8)		2 (12.5)
Prostate hypertrophy	2 (1.8)	-	-	2 (5.7)	-	-	1 (1.4)	1 (2.6)	-
Skin disease	2 (1.8)	1 (5)	1 (1.4)	1 (2.9)	-	1 (5.6)	1 (1.4)	1 (2.6)	-
Multiple sclerosis	1 (0.9)	-	1 (1.4)	-	-	-	1 (1.4)	-	-
Other	11 (9.9)	1 (5)	-	11 (31.4)	-	2 (11.1)	9 (12.3)	2 (5.3)	1 (6.3)
Total	111	20 (18)	74 (66.7)	35 (31.5)	2 (1.8)	18 (20)	73 (65.8)	38 (34.2)	16 (21.9)

* Rather than injection site pain.

**Table 5:** Effect of age, gender, vaccination status, history of laboratory-confirmed SARS-CoV-2 infection on anti-spike IgM and IgG antibody levels measured in healthcare workers [quantitative data are presented as number (%)].

Anti-Spike IgG Concentration (ng/mL)		Age Groups								Gender (N = 173)		COVID Infection-Symptoms (N = 165)			Booster Dose Vaccination (N = 172)		Symptoms after Vaccination-COVID-19 Infection (N = 162)			
		<21	21–30	31–40	41–50	51–60	61–70	>71–80	Total	F	M	No-No	Yes-Yes	Yes-No	No	Yes	Yes-Yes	Yes-No	No-Yes	No-No
	<1000	-	-	-	-	1 (3.3)	-	-	1 (0.6)	1 (0.9)	-	-	1 (2.2)	-	-	1 (9.6)	-	-	-	-
	1000–6000	-	1 (3.1)	1 (3.3)	6 (15)	5 (16.7)	3 (12.5)	2 (11.1)	18 (10.4)	15 (14)	3 (4.5)	16 (13.7)	2 (4.4)	-	6 (8.8)	12 (11.5)	-	8 (13.6)	1 (7.7)	7 (11.5)
	6000–11,000	1 (14.3)	5 (15.6)	7 (23.3)	3 (7.5)	8 (26.7)	8 (33.3)	1 (9.1)	33 (19.1)	21 (19.6)	12 (18.2)	25 (21.4)	6 (13.3)	-	13 (19.1)	20 (19.2)	4 (13.8)	12 (20.3	1 (7.7)	15 (24.6)
	11,000–16,000	3 (42.9)	5 (15.6)	8 (26.7)	14 (35)	9 (30)	3 (12.5)	4 (36.4)	46 (26.6)	29 (27.1)	17 (25.8)	37 (31.6)	6 (13.3)	1 (33.3)	21 (30.9)	24 (23.1)	2 (6.9)	18 (30.5)	4 (30.8)	18 (29.5)
	16,000–21,000	3 (42.9)	19 (59.4)	13 (43.3)	15 (37.5)	7 (23.3)	10 (41.7)	5 (45.5)	70 (40.5)	39 (34.4)	31 (46.9)	36 (30.7)	28 (62.2)	2 (66.7)	28 (41.2)	42 (40.4)	22 (75.9)	18 (30.5)	7 (53.8)	20 (32.8)
	21,000–26,000	-	2 (6.3)	1 (3.3)	2 (5)	-	-	-	5 (2.9)	2 (1.9)	3 (4.5)	3 (2.6)	2 (4.4)	-	-	5 (4.8)	1 (3.4)	3 (5.1)	-	1 (1.6)
	Total	7 (4)	32 (18.5)	30 (17.3)	40 (23.1)	30 (17.3)	24 (13.9)	11 (5.9)	173	107 (61.8)	66 (38.2)	117 (70.9)	45 (27.3)	3 (1.8)	68 (39.5)	104 (60.5)	29 (17.9)	59 (36.4)	13 (8)	61 (37.7)
Anti-Spike IgM Concentration (ng/mL)	<1000	-	-	-	-	-	-	-	-	-	-	-	-	-	-	-	-	-	-	-
	1000–2000	4 (57.1)	8 (25)	4 (13.3)	17 (42.5)	14 (46.7)	10 (41.7)	3 (27.3)	60 (34.7)	38 (35.5)	22 (33.3)	38 (32.5)	16 (35.6)	-	20 (29.4)	40 (38.5)	10 (34.5)	20 (32.9)	3 (23.1)	20 (3.3)
	2000–3000	3 (42.9)	13 (40.6)	15 (50)	15 (37.5)	14 (46.7)	7 (29.2)	2 (11.1)	70 (40.5)	41 (38.3)	28 (42.4)	50 (42.7)	15 (33.3)	2 (66.7)	30 (44.1)	38 (36.5)	10 (34.5)	25 (42.4)	6 (46.2)	24 (39.3)
	3000–4000	-	7 (21.8)	7 (23.3)	5 (12.5)	1 (3.3)	6 (25)	1 (9.1)	27 (15.6)	17 (15.9)	10 (15.2)	19 (16.2)	8 (17.8)	-	12 (17.6)	15 (14.4)	5 (17.2)	8 (13.6)	3 (23.1)	11 (18)
	4000–5000	-	3 (9.4)	3 (10)	2 (5)	1 (3.3)	1 (4.2)	2 (11.1)	12 (6.9)	6 (5.6)	6 (9.1)	7 (5.9)	4 (8.9)	-	4 (5.9)	8 (7.7)	3 (10.3)	3 (5.1)	1 (7.7)	5 (8.2)
	5000–6000	-	1 (3.1)	1 (3.3)	1 (2.5)	-	-	1 (9.1)	4 (2.3)	4 (3.7)	-	2 (1.7)	2.2	1 (33.3)	2 (2.9)	2 (1.9)	1 (3.4)	3 (5.1)	-	-
	12,000–13,000	-	-	-	-	-	-	1 (9.1)	1 (0.6)	1 (0.9)	-	1 (0.9)	-	-	1 (1.5)	-	-	-	-	1 (1.6)
	Total	7 (4)	32 (18.5)	30 (17.3)	40 (23.1)	30 (17.3)	24 (13.9)	11 (5.9)	173	107 (61.8)	66 (38.2)	117 (69.4)	45 (24.3)	3 (1.7)	68 (39.5)	104 (60.5)	29 (17.9)	59 (36.4)	13 (8)	61 (37.7)

## Data Availability

Data are available upon reasonable request by corresponding author.

## Supplementary Materials

The following supporting information can be downloaded at: https://www.mdpi.com/article/10.3390/vaccines10122038/s1, Table S1: title: Time distribution of first, second and third BNT162b2 vaccination among study participants [Quantitative data are presented as number (%)].
