# Supplementary material for: SARS-CoV-2 Specific Humoral Immune Responses after BNT162b2 Vaccination in Hospital Healthcare Workers
Source: Vaccines (Basel). 2022 Nov 29;10(12):2038. doi: 10.3390/vaccines10122038 (PMC9782529; doi:10.3390/vaccines10122038)
Supplement: Supplementary file 1 [file vaccines-10-02038-s001.zip › vaccines-2012614-supplementary.pdf]

Table S1. Time distribution of first, second and third BNT162b2 vaccination among study participants [Quantitative data are presented as number (%)].

Time of vaccination	First dose	Second dose	Third dose
December 2020	31 (18.2)	-	-
January 2021	74 (43.5)	57 (33.5)	-
Feburary 2021	15 (8.2)	38 (22.4)	-
April 2021	53 (30.6)	30 (17.6)	-
March 2021	-	48 (26.5)	-
September 2021	-	-	20 (19.2)
October 2021	-	-	31 (29.8)
November 2021	-	-	33 (31.7)
December 2021	-	-	20 (19.2)
Total	**173**	**173**	**104**
